# Supplementary material for: Designing a Multiplex PCR-xMAP Assay for the Detection and Differentiation of African Horse Sickness Virus, Serotypes 1–9
Source: Microorganisms. 2024 May 3;12(5):932. doi: 10.3390/microorganisms12050932 (PMC11124020; doi:10.3390/microorganisms12050932)
Supplement: Supplementary file 1 [file microorganisms-12-00932-s001.zip › microorganisms-2955301-supplementary.pdf]

Table S1. Different conditions tested for PCR optimisation of multiplex assay. Testing was performed using multiplex real-time PCR.

Condition	PCR additive	Primers heated to 95°C before addition of mastermix	Primer concentration (µM)	PCR annealing	PCR extension	Primer used in mastermix for AHSV serotypes 1-9[1]	Average normalised fluorescence[2]
1	x	x	0.2	60 °C		W,B,B,B,B,W,B,B,B	0.339
2	DMSO	x	0.2	60 °C		W,B,B,B,B,W,B,B,B	0.161
3	BSA	x	0.2	60 °C		W,B,B,B,B,W,B,B,B	0.318
4	DMSO + BSA	x	0.2	60 °C		W,B,B,B,B,W,B,B,B	0.114
5	x	x	0.1	60 °C		W,B,B,B,B,W,B,B,B	0.253
6	x	Yes	0.2	60 °C		W,B,B,B,B,W,B,B,B	0.757
7	x	Yes	0.2	55 °C	72 °C	W,B,B,B,B,W,B,B,B	0.766
8	x	Yes	0.2	70-60* °C	72 °C	W,B,B,B,B,W,B,W,B	0.726
9	x	Yes	0.2	65-55* °C	72 °C	W,B,B,B,B,W,B,B,B	1.071
10	x	Yes	0.2	65-55* °C	72 °C	W,B,B,B,B,W,B,W,B	1.336

[1]Letters denote primers used in multiplex mastermix for each serotype from either Bachanek-Bankowska et al. assay (B) or Weyer et al. assay (W)

[2]Fluorescent TaqMan probes and AHS RNA templates were included in the mastermix. The average normalised fluorescence was the deltaRN calculated using the ABI7500 software at the final cycle of the PCR, averaged for all nine serotypes. A higher value suggests greater amplification of the target nucleic acid.

*** Performed as touchdown PCR**

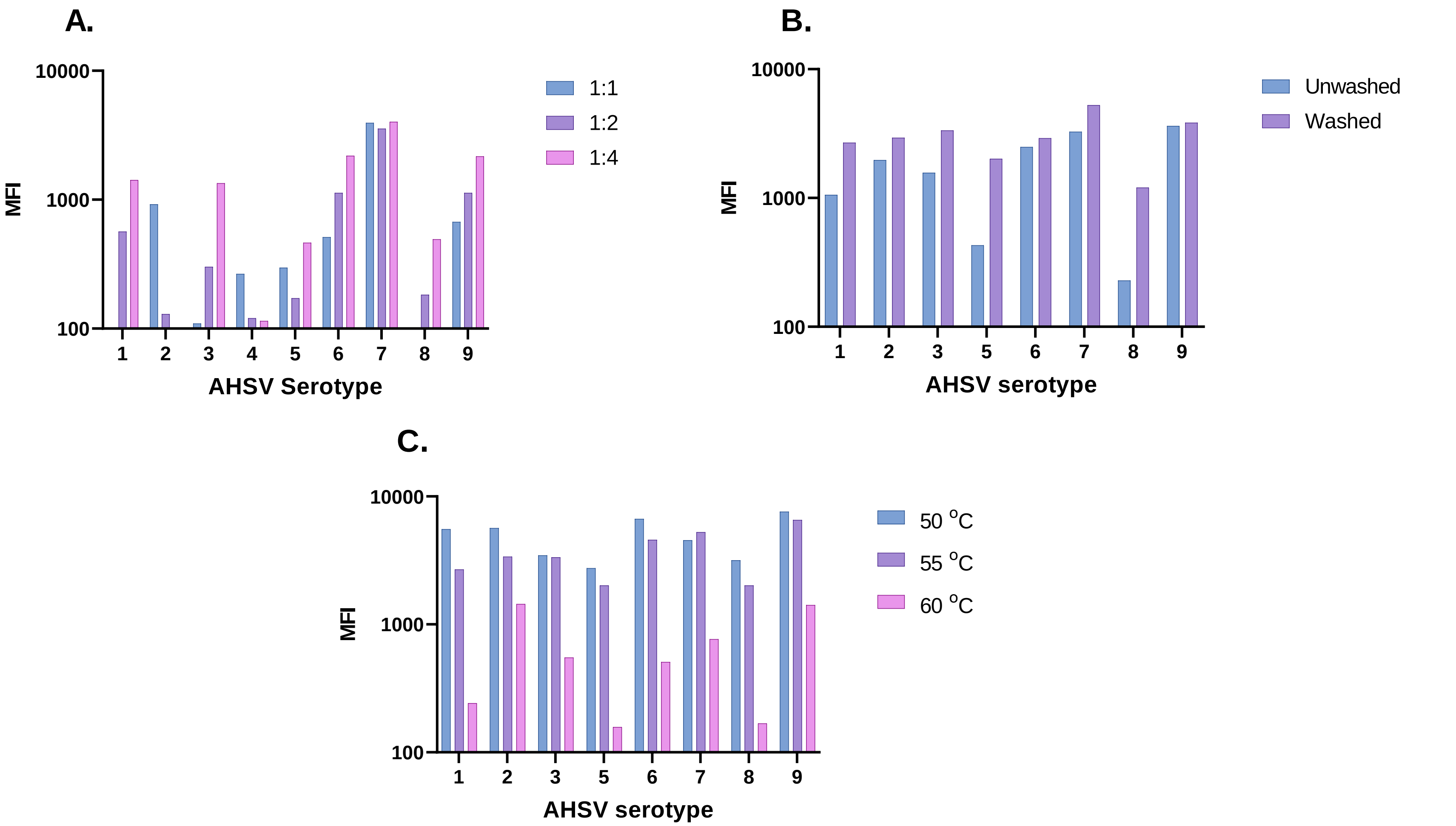

Figure S1 Different conditions tested for xMAP optimisation of multiplex assay. A. Primers were tested at either equimolar (1:1) or asymmetric concentrations (1:2, 1:4). (B) Assay was tested using the direct DNA hybridisation washed protocol or the direct DNA hybridisation: no wash protocol. (C) Different temperatures were tested for the microsphere-amplicon hybridization step of the xMAP assay.

Table S2. **The nine AHSV reference strains for serotypes 1-9 detected using the multiplex PCR-xMAP methodology. The median fluorescent intensity (MFI) of each duplicate sample is displayed.**

		AHSV detection in xMAP instrument (MFI)																	
Sample	Serotype	AHSV-1		AHSV-2		AHSV-3		AHSV-4		AHSV-5		AHSV-6		AHSV-7		AHSV-8		AHSV-9	
RSArah1/03	AHSV-1	2566.5	2887	0	0.5	-17.5	-10	44	27.5	4.75	5.75	26.75	13.25	38.75	51.75	25.75	12.25	26.25	3.25
RSArah2/03	AHSV-2	-32.5	-21.5	4066	4127	-2.5	35.5	62	40	18.75	1.75	30.25	28.75	5.75	30.75	28.75	33.75	50.75	16.25
RSArah3/03	AHSV-3	8.5	-7.5	68.5	34	3402.5	3226.5	25	39	35.75	31.75	45.75	48.75	65.75	23.75	16.75	33.75	67.25	34.25
SPArah4/03	AHSV-4	-23.5	-14	36	19	91.5	127.5	5941	5980.5	24.75	-1.25	58.75	21.75	28.75	56.75	35.75	11.75	52.25	23.25
RSArah5/03	AHSV-5	-31.5	-21.5	27.5	36.5	53	21	18	35	3408.75	2942.25	24.75	62.75	40.25	48.25	6.75	29.75	19.75	16.25
RSArah6/03	AHSV-6	121	-49.5	16	50	154.5	12.5	20	47	187.75	6.25	6886.25	7304.75	234.25	41.75	-1.75	11.75	55.25	42.25
KENrah7/03	AHSV-7	0	8.5	28	25	7.75	13.25	20	13.5	6.25	4.25	44.75	50.75	4488.5	4227	19.75	59.25	52.25	29.75
RSArah8/03	AHSV-8	216.5	87.5	14	28.5	471.5	407	0.5	47.5	-2.25	-28.25	7.25	19.75	5.75	1.25	3119.75	2914.75	18.25	28.25
PAKrah9/03	AHSV-9	-20	-4.5	336.5	323	88	36	-16	51	18.25	26.25	8.75	22.75	21.75	32.75	52.75	2.75	6340.75	7007.25

Table S3. Evaluation of the diagnostic sensitivity of the multiplex PCR-xMAP assay through screening 59 equine EDTA blood samples previously identified as AHSV RNA-positive by pan-AHSV RT-qPCR and using a serotype-specific RT-qPCR.

Sample	Pan-AHSV RT-qPCR (C_T value)	Serotype-specific RT-qPCR (AHSV serotype)	Multiplex PCR-xMAP (MFI)	Sample	Pan-AHSV RT-qPCR (C_T value)	Serotype-specific RT-qPCR (AHSV serotype)	Multiplex PCR-xMAP (MFI)
	Pos (30.35)	AHSV-6	AHSV-6 (2485)	31	Pos (20.54)	AHSV-4	AHSV-4 (2888)
2	Pos (21.54)	AHSV-6	AHSV-6 (6440)	32	Pos (24.08)	AHSV-9	AHSV-9 (6436)
3	Pos (30.86)	AHSV-6	AHSV-6 (2314)	33	Pos (19.16)	AHSV-2	AHSV-2 (1288)
4	Pos (24.72)	AHSV-6	AHSV-6 (5724)	34	Pos (18.80)	AHSV-2	AHSV-2 (3736)
5	Pos (23.93)	AHSV-6	AHSV-6 (5818)	35	Pos (25.03)	AHSV-9	AHSV-9 (9862)
6	Pos (30.83)	AHSV-6	AHSV-6 (2835)	36	Pos (22.82)	AHSV-2	AHSV-2 (2956)
7	Pos (22.31)	AHSV-6	AHSV-6 (7526)	37	Pos (24.18)	AHSV-2	AHSV-2 (2398)
8	Pos (24.87)	AHSV-6	AHSV-6 (6593)	38	Pos (21.02)	AHSV-2	AHSV-2 (878)
9	Pos (21.58)	AHSV-6	AHSV-6 (6930)	39	Pos (26.35)	AHSV-9	AHSV-9 (4948)
10	Pos (21.31)	AHSV-6	AHSV-6 (8404)	40	Pos (21.05)	AHSV-9	AHSV-9 (4345)
11	Pos (27.30)	AHSV-6	AHSV-6 (3880)	41	Pos (27.07)	AHSV-9	AHSV-9 (7532)
12	Pos (22.00)	AHSV-6	AHSV-6 (6982)	42	Pos (26.79)	AHSV-9	AHSV-9 (3449)
13	Pos (27.51)	AHSV-6	AHSV-6 (5167)	43	Pos (26.30)	AHSV-9	AHSV-9 (7477)
14	Pos (30.56)	AHSV-6	AHSV-6 (2521)	44	Pos (25.60)	AHSV-2	AHSV-2 (974)
15	Pos (27.77)	AHSV-6	AHSV-6 (4787)	45	Pos (26.97)	AHSV-9	AHSV-9 (5866)
16	Pos (24.32)	AHSV-6	AHSV-6 (6354)	46	Pos (23.94)	AHSV-9	AHSV-9 (6066)
17	Pos (26.09)	AHSV-6	AHSV-6 (5666)	47	Pos (23.00)	AHSV-9	AHSV-9 (9189)
18	Pos (27.21)	AHSV-6	AHSV-6 (4782)	48	Pos (26.24)	AHSV-9	AHSV-9 (7656)
19	Pos (36.20)	AHSV-6	AHSV-6 (4482)	49	Pos (25.64)	AHSV-9	AHSV-9 (7623)
20	Pos (38.02)	AHSV-6	AHSV-6 (1765)	50	Pos (28.95)	AHSV-2*	AHSV-2 (301)
21	Pos (19.29)	AHSV-4	AHSV-4 (4345)	51	Pos (23.61)	AHSV-5*	AHSV-5 (2931)
22	Pos (30.60)	AHSV-5	AHSV-5 (3111)	52	Pos (26.66)	AHSV-5*	AHSV-5 (838)
23	Pos (22.96)	AHSV-4	AHSV-4 (1656)	53	Pos (30.30)	AHSV-5*	AHSV-5 (221)
24	Pos (23.03)	AHSV-5	AHSV-5 (3083)	54	Pos (25.28)	AHSV-1*	AHSV-1 (1716)

25	Pos (25.13)	AHSV-4	AHSV-4 (7128)	55	Pos (25.82)	AHSV-1*	AHSV-1 (1589)
26	Pos (18.88)	AHSV-4	AHSV-4 (4643)	56	Pos (28.38)	AHSV-2*	AHSV-2 (525)
27	Pos (22.98)	AHSV-4	AHSV-4 (1248)	57	Pos (23.15)	AHSV-5*	AHSV-5 (2789)
28	Pos (22.25)	AHSV-5	AHSV-5 (2924)	58	Pos (26.91)	AHSV-5*	AHSV-5 (2097)
29	Pos (18.84)	AHSV-4	AHSV-4 (6141)	59	Pos (30.00)	AHSV-5*	AHSV-5 (273)
30	Pos (18.53)	AHSV-4	AHSV-4 (10218)				

*AHSV serotype data obtained from the organiser of the PT scheme, from which the samples were obtained.

Table S4. Eight equine EDTA blood samples which demonstrated discordance in the detection of AHSV using the pan-AHSV RT-qPCR and multiplex PCR-xMAP assay.

Sample ref.	Pan-AHSV RT-PCR (C_T value)	Serotype specific RT-PCR	Multiplex PCR-xMAP (MFI)
60	Pos (38.80)	AHSV-6	Not detected
61	Pos (39.73)	Not detected	Not detected
62	Pos (38.75)	AHSV-6	Not detected
63	Pos (22.07)	AHSV-4	Not detected
64	Pos (24.07)	AHSV-5	Not detected
65	Pos (28.28)	AHSV-5	Not detected
66	Pos (24.86)	AHSV-4	Not detected
67	Pos (25.71)	AHSV-1*	Not detected

*Serotyping results obtained from the organiser of the PT scheme, from which the samples were obtained.
